# The difference between horizontal-to-vertical spectra ratio and empirical transfer function as revealed by vertical arrays

**DOI:** 10.1371/journal.pone.0210852

**Published:** 2019-01-30

**Authors:** Mianshui Rong, Hongguang Li, Yan Yu

**Affiliations:** 1 Division of Engineering Seismology, Institute of Crustal Dynamics, China Earthquake Administration, Beijing, China; 2 Division of EarthquakeEngineering, National Earthquake Response Support Service, China Earthquake Administration, Beijing, China; Northeastern University, UNITED STATES

## Abstract

The horizontal-to-vertical spectral ratio (HVSR) and empirical transfer function analyses were performed on the S-wave recordings from two vertical borehole strong motion arrays: the Garner Valley Downhole Array in southern California, and the KiK-net Ichinoseki-Nishi Vertical Array in West Ichinoseki, Japan. The results show that the discrepancy between the HVSR and the transfer function is mainly caused by the significant site response of the vertical component, thus, vertical incident P-waves are proposed to play an important role in the vertical amplification. The P-wave amplification is frequency-dependent. In the low-frequency range within *f*_0_ (the fundamental frequency of the site), the effect of the vertical P-wave amplification is slight, this is why HVSR and transfer function match in this frequency range. In the high-frequency range near 2 *f*_0_ or larger, the P-wave amplification is obvious, which maybe explain the discrepancy between the HVSR and the transfer function.

## Introduction

The evaluation of site-effects due to local geology or topography has become a standard requirement in microzonation studies or site evaluation for important facilities [1]. Many empirical methods such as the standard spectral ratio method [2],the linear inversion method [3],the reference event method [4],and the HVSR method [5] are used to identify site characteristics.Among these methods, the HVSR is the spectral ratio technique using records of only one station. So it is an attractive low-cost method.

The HVSR method was initially used to estimate the fundamental period and was subsequently extended to determine shear-wave velocity of near-surface soils using ambient-noise/microtremor measurements [6–7]. However, it was found that results in the latter case can be ambivalent [8–9]. As Bonnefoy-Claudet and coworkers pointed out, ambient vibrations sources are (1) controlled by local surface sources, and (2) caused by the ellipticity of fundamental Rayleigh waves. Actually, the ambient wave field is largely dominated by surface waves [9]. Moreover, the peak frequency of HVSR under ambient vibrations can also be controlled by the peak frequency of Love waves, not only by Rayleigh waves [10]. The S-wave resonance of sediments is the main cause of site-effects in earthquake engineering. Recently, the HVSR method has been used extensively to estimate site-effects using accelerograms, whereby several publications compared HVSR with traditional methods such as spectral ratios or generalized inversions of the S-wave spectra of the horizontal components [1,11–16]. These comparative studies have shown that estimates of the fundamental frequency are similar to those obtained with traditional spectral ratios, but the amplitude level is different. Bonilla et al. [15] studied the borehole response at the Garner Valley Downhole Array and found that the discrepancy in estimated amplitude of the site response between HVSR and traditional spectral ratio is caused by the vertical component, which has significant site responses associated with it, due to S-to-P conversions that begin in the weathered granite boundary at 87m depth. Since that work, few studies explored in depth the difference between HVSR and traditional spectral ratio in amplitude of the site response for other cases. As pointed out by Theodulidis et al. [17], the traditional spectral ratio technique is the standard method for characterizing site amplification. It requires a pair of instruments, one located at the site under investigation and the other located at a reference site, but in many cases it is difficult to identify the ideal reference site. In recent years, the accumulated recordings of downhole arrays have provided a significant step forward to overcome the “reference site” hurdle. That the bedrock borehole ground motion can be used as the reference motion was proven by Steidl et al. [18], but the effect of the downgoing wave field and the resulting destructive interference must still be considered. Based on vertical borehole arrays, the traditional spectral ratio can be used as a good site effect indicator to further study those conditions that allow HVSR to be applied to determination of the site amplification factors.

In this article, strong motions recorded by two vertical borehole arrays,the Garner Valley Downhole Array (GVDA) and the KiK-net Ichinoseki-Nishi Vertical Array (IWTH25), are analyzed. First, the discrepancy between HVSR and transfer functions are revealed and discussed. Second, the 1-D seismic response method which can deal with site response under the excitation of P or SV waves in frequency domain and the wave number integral method used for construction of synthetic seismograms are applied to interpret site specific wave propagation characteristics. Finally, the applicability of HVSR on site response is explored.

## Methodology

### Horizontal-to-vertical spectral ratio (HVSR) method

The HVSR method was originally used to estimate the characteristics of the ground motion from the employment of the microtremor observation [5].Then it has been applied to weak and strong motions from earthquakes [1,19–20].The HVSR is defined as the ratio between the horizontal Fourier amplitude of ground motion, *H*_*s*_, and the vertical Fourier amplitude of ground motion, *V*_*s*_, at the free surface:

$$HVSR=\frac{H_{S}}{V_{S}}$$ (1)

Lermo and Chavez-Garcia [1] found that the HVSR of the S-wave part of a strong-motion record can be used to estimate transfer function empirically. Kawase et al. [21] proved that HVSR can be calculated as the amplitude ratio between transfer functions for the horizontally polarized S-wave incidence and the vertically polarized P-wave incidence, both calculated at the observation point with a coefficient depending on the bedrock property. Based on the diffuse field theory, the HVSR method can be used to invert a 1D velocity structure through microtremors [22] or earthquake recordings [21]. Rong et al. [23] ever compared HVSRs of the S-wave with 1-D equivalent-linear numeral simulation on 21 station sites in western China and suggested that the HVSR from observed earthquake ground motion resembles the empirical transfer function of nonlinear site-response. Here we present a comparison study between HVSRs and transfer functions.

### Empirical transfer function

Since introduction of the Empirical Transfer Function [2], many studies have used the spectral ratio approach to estimate site response, traditionally by comparing ground motions at sites of interest to a nearby rock site that is considered a reference motion. According to the traditional spectral ratio method, a seismogram can be calculated as the convolution of the source, path, site effect, and instrument response as:

$$A_{ij}(f)=S_{i}(f)P_{ij}(f)G_{j}(f)I_{j}(f)$$ (2)

where *S*_*i*_*(f)* is the source term of the *i*th event, *P*_*ij*_*(f)* is the path term between the *j*th station and the *i*th event, *G*_*j*_*(f)* is the site term for the *j*th station, and *I*_*j*_*(f)* is the instrument response term for the *j*th station. The spectral ratio is obtained by dividing the Fourier spectrum of the acceleration at the *j*th station by the spectrum at the *k*th reference station as follows:

$$\frac{A_{ij}(f)}{A_{ik}(f)}=\frac{S_{i}(f)P_{ij}(f)G_{j}(f)I_{j}(f)}{S_{i}(f)P_{ik}(f)G_{k}(f)I_{k}(f)}=\frac{G_{j}(f)}{G_{k}(f)}$$ (3)

If the distance between stations *j* and *k* is much less than their hypocentral distances from the source, the source and path term would be eliminated. The instrument response can be removed from the data under the assumption that both instruments are the same. The exact site response can thus be obtained from formula (3). The physical principle of the traditional spectral ratio method is obvious and it can identify reliably the predominant frequency of a site very well, but the common assumption that a nearby rock site represents the reference motion to a soil site can be questioned, as even surface rock sites located on competent crystalline have a non-flat site response which is most likely due to the weathered and fractured nature of the near surface that causes the velocity to drop. If the effect of downgoing wave fields and the resulting destructive interference are being considered, the bedrock borehole can be taken as a reference [18]. The borehole rock motion of vertical array sites (at a depth proven to be sufficient) was chosen as a reference site in our study. The traditional spectral ratio, *H_S_*/*H_B_*, is defined as Transfer Function (hereafter referred to as the TF) in this study. The *Hs* and *H*_*B*_ are the horizontal Fourier amplitude motions recorded on an alluvium site and on the basement respectively.

## The vertical array sites and data used

The increase in the number of borehole instruments provided a significant step forward in directly measuring the effects of surface geology and critical constraints on our methods for interpreting surface observations [15]. Borehole measurements provided direct *in situ* evidence for the research of the seismic response theory, method and applicability of this geotechnical model. In recent years, many downhole arrays have been built all over the world, and seismic records of two vertical arrays, GVDA and the IWTH25, were incorporated in our dataset.

**Fig 1:**
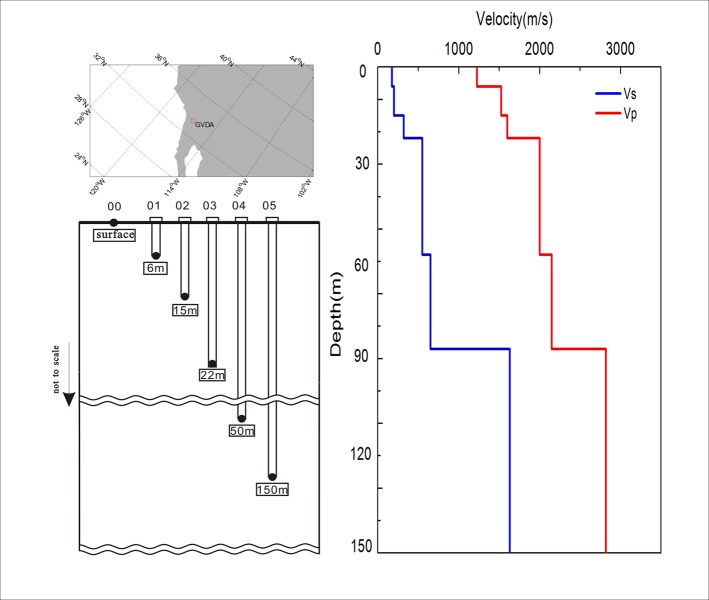
Locations of the GVDA site (a) and the schematic layout of its instruments (b) and velocity profiles (c).

The GVDA is a ground motion research site in a seismically active region in Southern California, US, maintained by NEES (http://www.nees.org/)(**Fig 1A**). The main Garner Valley station is instrumented with several downhole accelerometers (from 6 m sub-surface down to 150 m depth) (**Fig 1B**). The near-surface soil types present are silty sand, sand, clayey sand, and silty gravel. The alluvium gradually transitions into decomposed granite at depths between 18 m and 25 m. Decomposed granite consisting of gravely sand exists between 25 and 88 m. At a depth of 88 m the contact with granodiorite of the Southern California Peninsular Ranges batholith is reached. Bonilla [15] studied the GVDA velocity structure by computing synthetic accelerograms for a small event located at an epicentral distance of 10 km. His best final velocity model is adopted in our study, which is also presented in **Fig 1C**.

The aim of this paper is to further check the use of HVSR as an indicator of site-effects. In order to obtain a high signal-to-noise ratio, the seismic events which can trigger PGA greater than 5 cm/s^2^ within 300 km from the GVDA site, recorded between 1 January 2004 and 31 October 2015 were analyzed. There are twenty seismic events in total and all of them have been used to study the applicability of HVSR on site-effects [24].We directly use the same dataset as shown in the work of Rong et al. [24].

**Fig 2:**
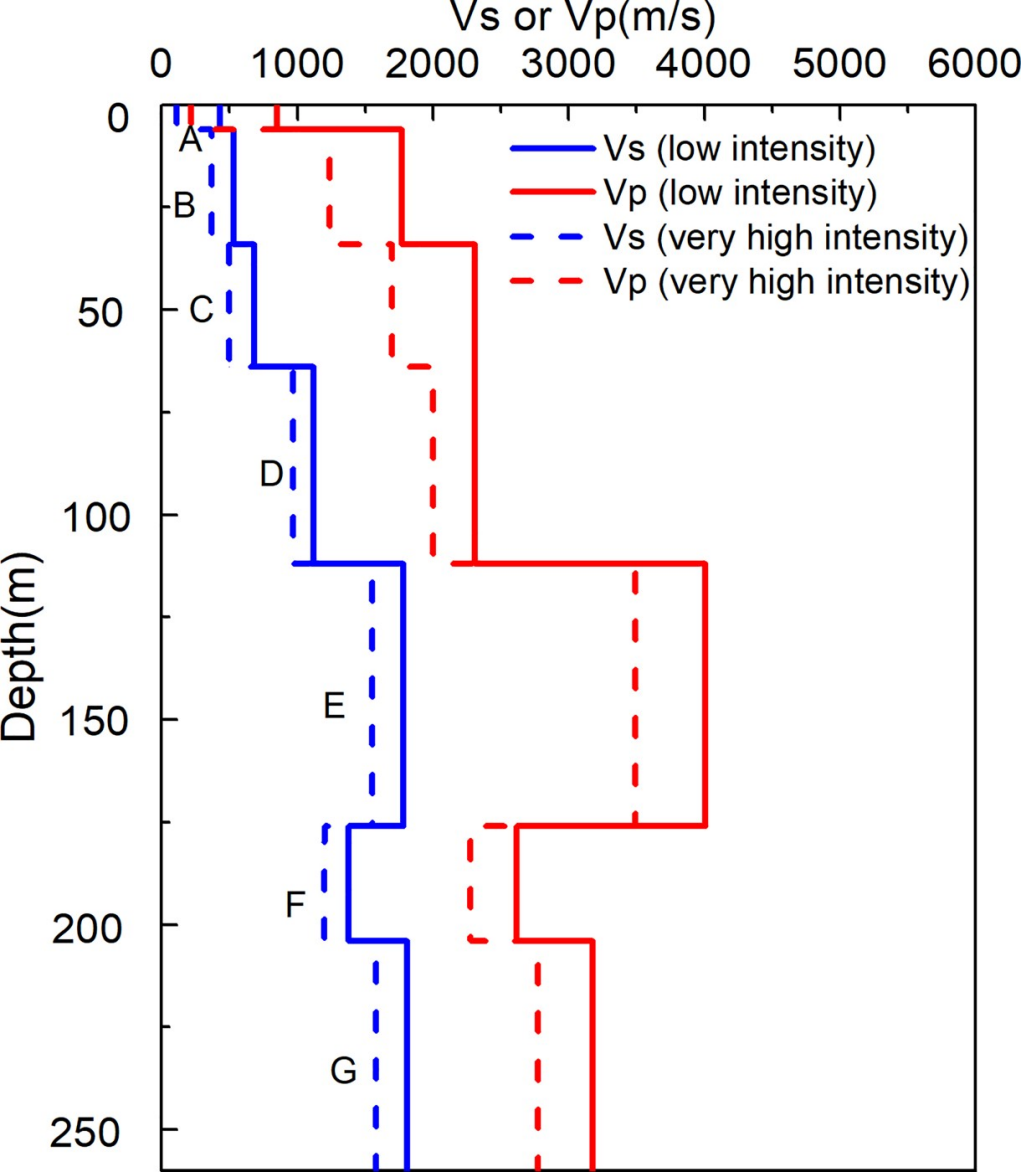
S- and P-wave velocity profiles at IWTH25.

The KiK-net Ichinoseki-Nishi Vertical Array (IWTH25) Site is one of the KiK-net stations, which is equipped with three-component accelerometers, installed at the surface and the bottom of a 260m borehole. Its velocity profile is presented in **Fig 2** and **Table 1**. It is well known that when the sites suffer very high intensity, the soil medium will exhibit nonlinear characteristics and its velocity changes greatly, as is shown in **Fig 2** and **Table 1**. This change of velocity will cause a shift in the predominant frequency and corresponding transformation of the HVSR curves. In order to facilitate a comparison of HVSR and TF, we limited our study to conditions approaching linear elastic status, which included 22 events with hypocentral distances within 100km and PGA 4-5gal as listed in **Table 2**.

**Table 1:** Velocity profile of IWTH25 [26].

Notes of strata	Density(t/m^3^)	Depth(m)	Low intensity		Very high intensity	
			Vs(m/s)	Vp(m/s)	Vs(m/s)	Vp(m/s)
A	1.6	6	430	850	110	217
B	1.6	34	530	1770	370	1236
C	1.6	64	680	2310	500	1699
D	1.7	112	1120	2310	970	2001
E	2.0	176	1780	4010	1550	3492
F	1.8	204	1380	2620	1200	2278
G	2.1	260	1810	3180	1580	2776

**Table 2:** Earthquake parameters of the accelerograms used for IWTH25.

No.	Time(UTC)	M	Depth(km)	Epi. Dis.(km)	Long.	Lat.	PGA(cm/s^2^)
1	12/10/2000 21:30:25	4.0	79	48	141.40	39.13	4.6
2	08/24/2002 08:22:30	4.3	15	93	141.32	38.25	4.8
3	11/16/2002 12:20:06	4.2	7	40	141.13	38.71	4.1
4	05/26/2003 18:29:48	4.4	63	71	141.65	38.83	4.7
5	05/26/2003 21:33:11	4.0	74	65	141.59	38.87	4.4
6	06/09/2003 03:58:17	4.1	64	72	141.70	38.95	4.7
7	06/22/2003 12:33:55	4.4	74	65	141.58	38.82	4.7
8	10/02/2003 16:32:43	4.0	88	51	141.44	39.09	4.8
9	11/02/2003 09:36:21	4.0	74	66	141.60	38.87	4.0
10	03/26/2004 00:01:13	4.0	71	67	141.64	38.95	4.5
11	06/28/2004 11:23:57	4.1	76	66	141.61	38.88	4.9
12	10/18/2004 17:00:52	3.7	73	67	141.62	38.93	4.9
13	01/26/2006 03:00:34	3.8	68	70	141.63	38.83	4.0
14	03/24/2006 07:59:24	4.0	74	67	141.59	38.82	4.8
15	06/11/2006 05:22:30	3.1	6	26	140.57	39.05	4.6
16	09/05/2006 07:10:04	3.8	72	68	141.64	38.91	4.9
17	04/17/2008 04:20:00	5.8	166	55	140.23	39.04	4.8
18	05/29/2008 01:42:03	4.8	1	25	140.58	38.99	4.1
19	06/15/2008 04:23:32	2.7	3	23	140.79	38.81	4.1
20	06/17/2008 06:39:31	4.1	3	25	140.80	38.79	4.6
21	06/20/2008 02:32:13	3.9	10	20	140.97	39.17	4.7
22	08/13/2008 21:19:11	3.4	73	70	141.61	38.78	4.0

## Comparison of HVSRs and TFs

**Fig 3:**
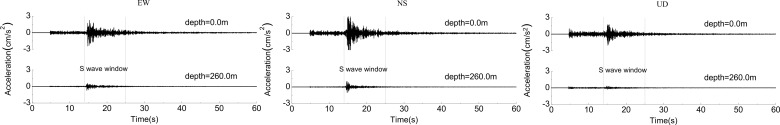
Time histories at different depths of IWTH25 site during the M_L_ 4.0 earthquake of 10 December, 2000.

HVSRs can be obtained through strong motions recorded by the sensors located at different depths for every seismic event. **Fig 3** presents an example of observations at different depths for the three components of ground acceleration from IWTH25 site. When the seismic waves propagate from the bedrock through the soil column, the surface to bedrock amplification is significant. The HVSR curves are determined using spectral ratio of S-wave part of horizontal and vertical components. The TF curves are determined using spectral ratio of S-wave part of components at surface over incident upgoing S-wave at borehole site. A more than 10s window beginning 0.5–1 sec before the onset of the S-wave is taken from each records. A 5% Hanning taper was applied to all time windows. All the S-wave Fourier spectra are smoothed by using the logarithmic smoothing function proposed for this correction [25]. Once the spectral ratio for each station and each earthquake was obtained, the logarithmic average and the ±1 standard deviation or the 95% confidence limits of the mean were calculated.

**Fig 4:**
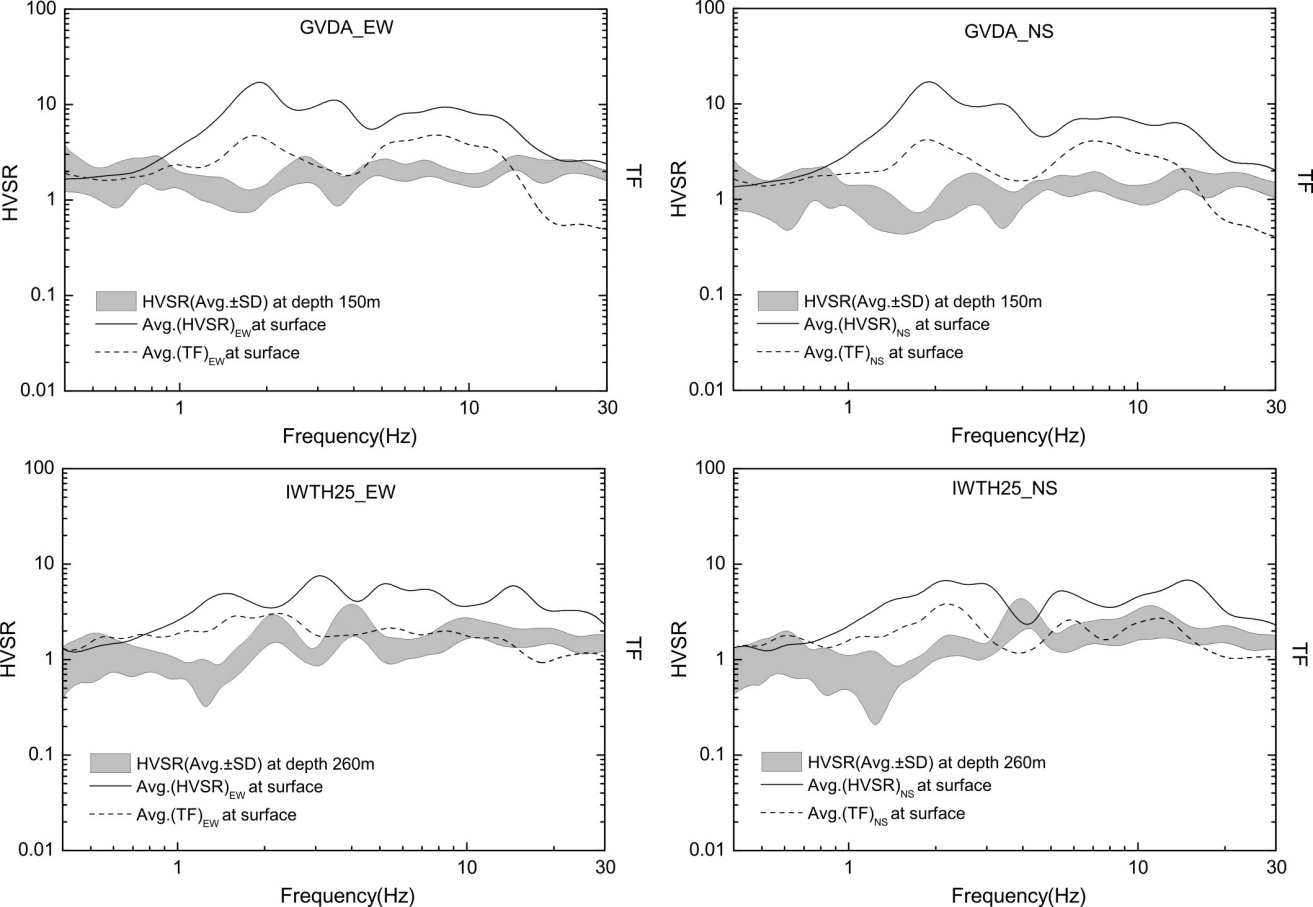
HVSRs and TFs of seismic events recorded by GVDA and IWTH25.

Considering the difference of HVSR derived from different components, we calculated HVSRs through different components separately. For the GVDA and IWTH25 site, these are EW/UD and NS/UD. **Fig 4** respectively shows HVSRs and TFs at different depths for two vertical arrays. In this Figure the shaded areas constitute the average of results of all the corresponding considered events with ±1 standard deviation, while the TFs are evaluated using traditional spectral ratios of the S-wave, as explained above.

As we can see from **Fig 4**, firstly, the HVSRs and TFs on the surface of sites yield quite similar results in the whole considered frequency band. They have the same spectrum shapes. The predominant frequencies obtained from HVSRs are in good agreement with TFs. Since it has been recognized that the TF is a reasonable indicator of site-effects, whose peak frequency usually is the predominant frequency of a site, the agreement of spectrum shapes between HVSRs and TFs further verified the applicability of HVSR in pinpointing the resonant frequency, as previous studies proved before [11–14]. In our cases, the predominant frequency of GVDA site is about 1.8 Hz, coincident with Archuleta’s [27] research results, the predominant frequency of IWTH25 is about 2.0Hz. Secondly, HVSRs and TFs have different amplitudes, commonly, the amplitudes of TF exceed that of HVSRs in the whole considered frequency band. Thirdly, the HVSRs at the bedrock are relatively flat, their values can be seen as varying around a constant, but for different sites, the extent of the fluctuation is different. According to Nakamura’s basic assumption, the firm substrate propagation is even in all directions. Obviously, the GVDA site is in accordance with this assumption, the HVSRs at the depth of 150m is close to 1. For the IWTH25 site, the HVSRs at the depth of 260m vary around 1, the maximal amplitude is about 3 near 4 Hz, that fluctuation is likely due to the sudden change of velocity from 176m to 204m, where bedrock discontinuity appears.

## Interpretation of the observations

**Fig 5:**
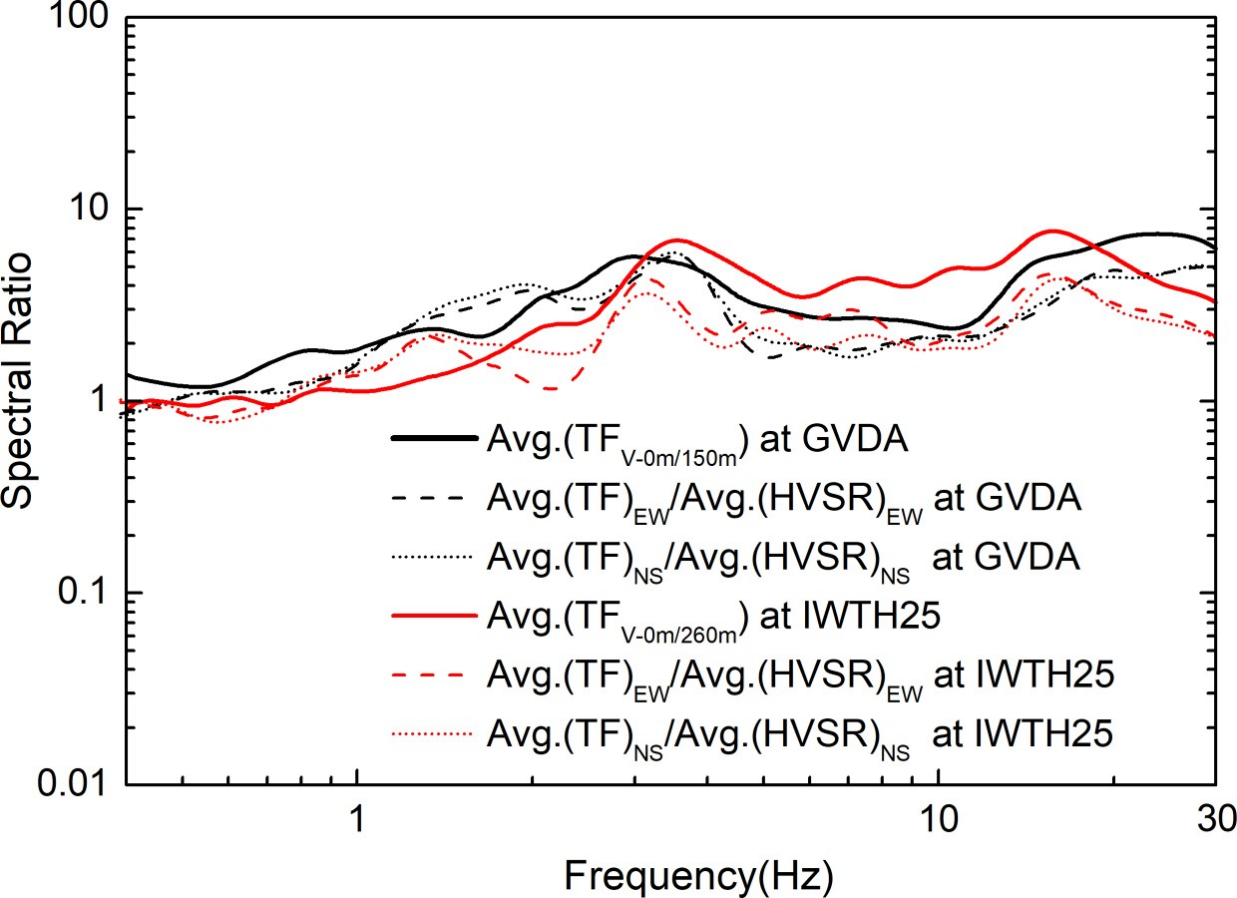
Average TF from S-wave in vertical components, taken the borehole bedrock as the reference site, and the ratios of average TFs to average HVSRs (dashed or dotted lines) for the EW and NS components.

In order to interpret the amplitude discrepancy between the HVSR and the TF for different arrays, the ratio of the average TF to the average HVSR is introduced, as shown in Fig 5. According to the definition of the HVSR and TF, the ratio can be written as (*H_S_*/*H_B_*)/(*H_S_*/*V_S_*) = *V_S_*/*H_B_*. Under the assumption that the HVSR in the firm substrate could be regarded as a constant, the shape of the ratio *V_S_*/*H_B_* is consistent with the vertical TF, which is denoted by the ratio *V_S_*/*V_B_*. The TFs of the GVDA and IWTH25 are also shown in Fig 5. As shown in Fig 5, we see the ratios of the average TFs to the average HVSRs are in good agreement with the average vertical TF, indicating that the spectral discrepancy between the TF and HVSR is mainly controlled by the site response of the vertical component.

Kawase et al. [21] proved that HVSR can be calculated as the amplitude ratio between TF for the horizontally polarized S-wave incidence and the vertically polarized P-wave incidence, both calculated at the observation point using a coefficient depending on the bedrock property. Their conclusions were based on the diffuse field theory for plane body waves. Nakamura [5] also believed that the tremor can be divided into a horizontal and a vertical direction, whereby the horizontal tremor can be considered to be amplified through multi-reflection of the S-wave, while the vertical tremor is through multi-reflection of the P-wave. Actually, in ideal conditions of vertical incidence there are no P-SV conversions at the discontinuities, so the vertical component will only be induced by vertical incident P-waves. Generally, the major vertical amplification character can be obtained using P-wave as basement excitation, although a discontinuous, inhomogeneous medium would complicate wave propagation due to multiple scattering. To further interpret the vertical amplification effect revealed by **Fig 5**, the transfer matrix method [28] was applied to calculate the seismic response in a linear regime. For this the velocity models of **Fig 1C** and **Table 1** were adopted. Considering the downgoing wave effect, we induced the borehole response and outcrop response, as proposed by Bonilla et al. [15]. The borehole response is the observed spectrum ratio of records at surface over records at borehole site. The outcrop response is the observed spectrum ratio of records at surface over incident wave at borehole site. The borehole response is the site response indicator taking into account the downgoing wave effect; and the outcrop response is the corresponding index eliminating the downgoing wave effect.

**Fig 6:**
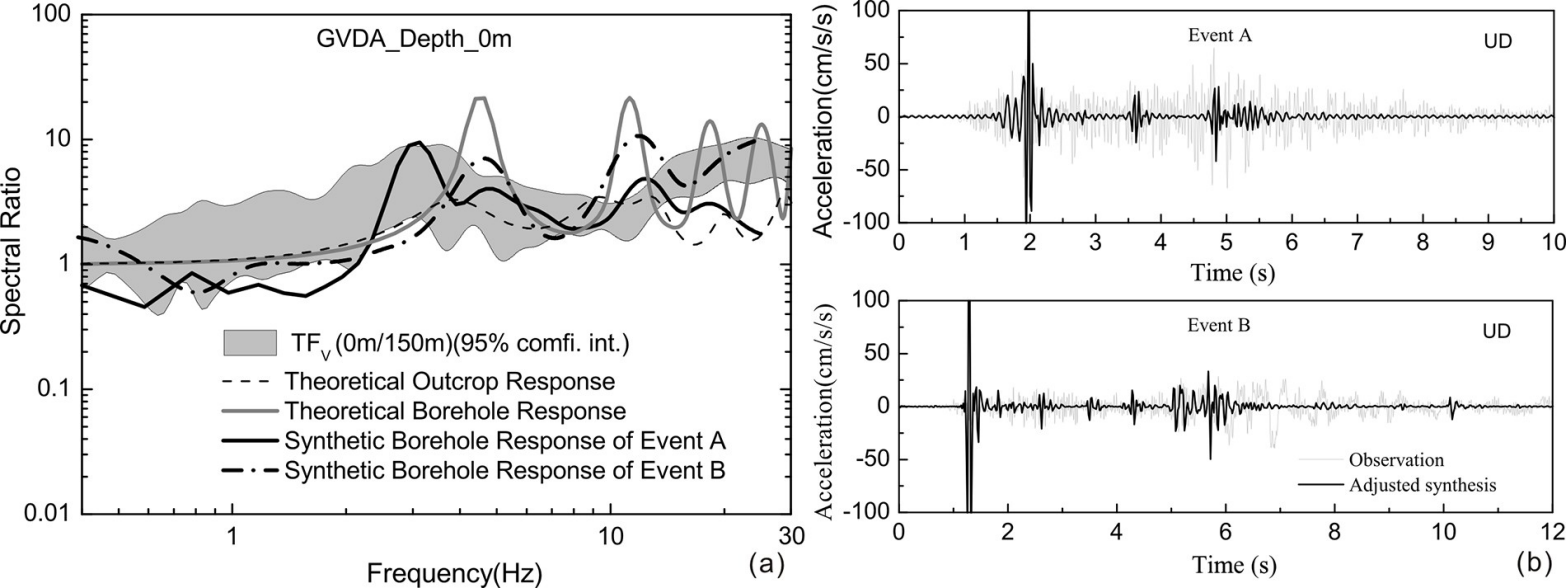
Borehole response, outcrop response and vertical site response of GVDA (a) and observed and synthetic accelerograms of two earthquakes.

In **Fig 6A**, the spectral shapes of vertical seismic responses at surface are shown for the GVDA. Also shown in **Fig 6A** are the theoretical and synthetic borehole response, and the theoretical outcrop response. The peak frequencies of the theoretical borehole response of GVDA are about 4.5 Hz, while corresponding peak frequencies of outcrop response are about 4 Hz, closer than the observed peak value of 3 Hz. It has to be mentioned that the theoretical borehole and outcrop response are obtained under the condition of vertical incident P-waves in a linear regime, ignoring any effect of material damping and incident angle. To consider possible effects of these two factors, the wave number integral method [29] is introduced to compute synthetic accelerograms based on velocity profiles proposed by Bonilla [15]. The focal characteristics of two selected events (the M_L_ 5.4 earthquake of 7 July 2010 and the M_L_ 5.4 earthquake of 12 June 2005), which are offered by Harvard University (http://www.globalcmt.org/) are presented in **Table 3**. The synthetic borehole responses in **Fig 6A** are derived from a P-wave window of vertical component in **Fig 6B**. The amplitudes of synthetic accelerograms of these two events are much bigger than the observation, so we adjust the amplitudes of synthetic accelerograms and put them together with the observations. Anyway, the comparison of the shape of synthetics and observed time histories shows that the P waves explain the largest part of the vertical amplification. The synthetic borehole responses are in good agreement with observed vertical transfer function, especially for event A, which further verify that the vertical amplification can be explained by the borehole response of incident P-waves.

**Table 3:** Focal parameters of two earthquakes used to synthetize.

Event	Date(yymmdd)	Time(hh:mm:ss)	M_L_	Latitude	Longitude	Depth(km)	Strike	Dip	Rake	Event ID
A	2005-06-12	15:41:46	5.2	33.530	-116.570	14	305	53	-179	14151344
B	2010/07/07	23:53:34	5.4	33.420	-116.489	14	318	83	-179	10736069

## Discussion of conditions for using HVSR as TF

According to analysis in previous section, based on observation, it is clear that we cannot always assume a borehole sensor located below the soil column in competent granite rock **as** a suitable reference site, as defined by Steidl et al. [18], with a flat amplitude response in the frequencies of engineering interest. The HVSRs revealed by instruments located on 260m (soft sandstone with *V*_*s*_ 1810m/s) of the IWTH25 site indicates that at this depth, the site may undergo amplification that cannot be ignored. It also appears that one of the principle assumptions that H/V is equal to unity on the bedrock does not necessarily apply in all circumstances.

**Fig 7:**
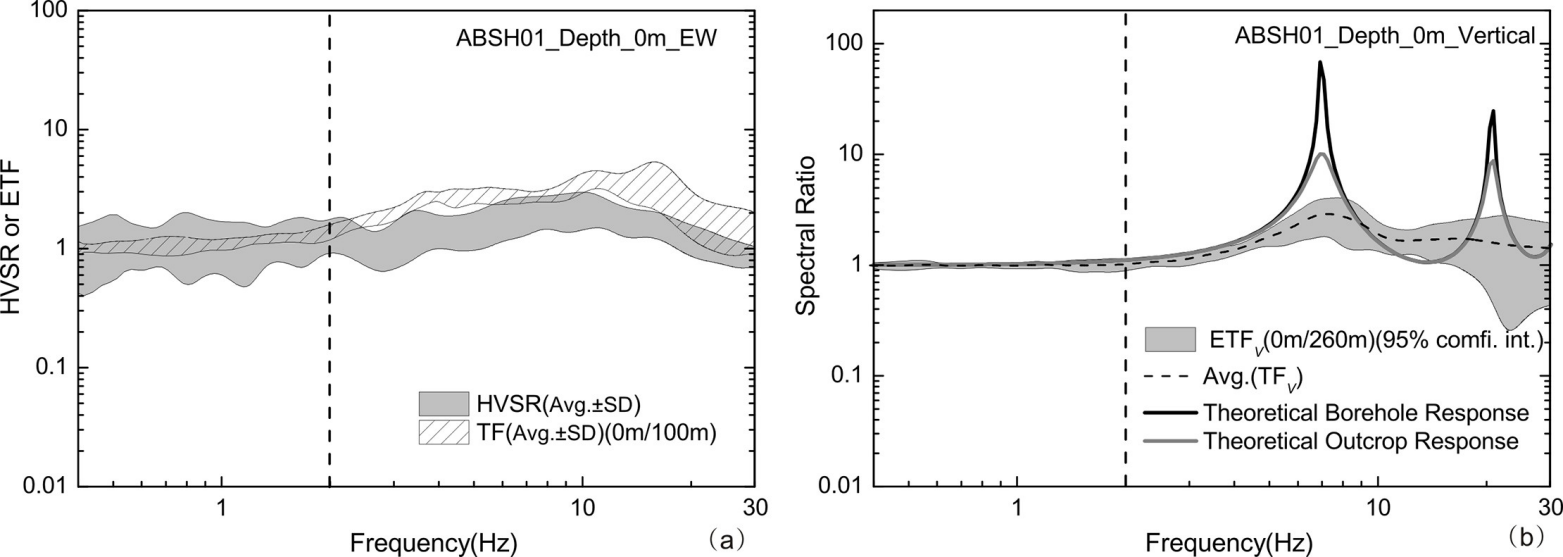
HVSRs (shaded area) and TFs (shaded area with oblique lines) of seismic events recorded by ABSH01 (a) and its Borehole response, outcrop response and vertical site response (b).

We compared HVSR and TF for the two sites (GVDA and IWTH25), and proved that their differences are based on vertical site responses, through theoretical solution of wave propagation in elastic layered site and synthetic accelerograms, which revealed that the vertical amplification caused by the vertical incident P-wave is probably the main reason for the vertical site responses. This result demonstrates that the other principle assumption of the HVSR method, namely that “the vertical motion is not significantly amplified by the surface layers”, is not generally applicable.However, there remains no doubt that HVSR resembles TF for the two vertical arrays, as the shapes of HVSRs agree quite well with TFs, which further confirmed its validity in determining the predominant frequency of a site. The discrepancy between HVSR and TF is closely related to the level of the vertical P-wave amplification, and the amplification is frequency dependent. Commonly, the P*-*wave wavelength is about two times larger (depending on the Poisson ratio) than the S-wave wavelength. The fundamental frequency of the site can be evaluated using the equation *f*_*0*_ *= Vs/4h*, the fundamental frequency under the excitation of P-wave should be about 2 *f*_*0*_. Generally, in the low-frequency range, the P-wave wavelength is much larger than the depth of soil strata, so the size of the soil column can be ignored compared to the P-wave wavelength. As the frequency increases, the P-wave wavelength reduces and becomes comparable to soil profiles, so that the effect of the soil strata becomes more obvious. In our study, it is obvious that in the low-frequency range within *f*_0,_ the level of the vertical P-wave amplification is slight, while in high-frequency range near 2 *f*_0_ or larger, the P-wave amplification is obvious. Based on these considerations, we can deduce that HVSRs and TFs should be almost exactly the same for those frequency ranges in which the P-wave amplification can be completely ignored. We casually selected ABSH01 from many KiK-net borehole arrays and analysed 14 events with PGA 1.1–9.5cm/s^2^, recorded between 26 September 2003 and 15 May 2015. **Fig 7** presents the obtained TF, HVSR and vertical response. In the range of 0.4-2Hz, there is no P-wave amplification, as shown in **Fig 7B**. So we can deduce that in this frequency range, the observed HVSR should be consistent with the TF, which indeed verified by **Fig 7A**.

**Fig 8:**
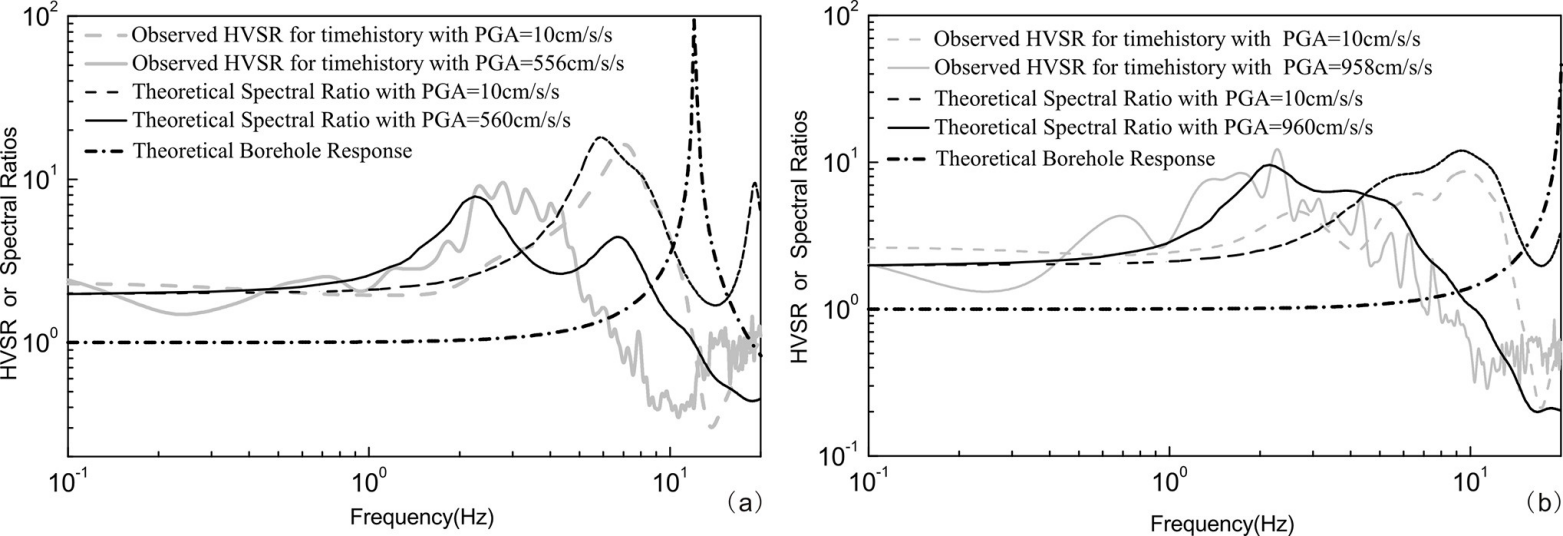
The comparison of the HVSRs (grey lines) of S-wave motions and the Theoretical Spectral Ratios (black lines) of S-waves from 1-D equivalent-linear models at several PGA levels for stations 51SFB (a) and 51WCW (b) (adapted from [23]) and the theoretical Borehole Responses of these two sites.

In our dataset, most of observed PGAs of the considered events for two vertical arrays are less than 50cm/s^2^, whereas only two events (recorded by GVDA) have PGAs > 50cm/s^2^, so the performance of these sites is closer to a linear regime. However, a similar conclusion can be obtained even considering the site nonlinearity under severe earthquakes. We take two stations in western China as examples. Rong et al. [23] conducted HVSR analysis using the strong motion records of the Wenchuan Ms8.0 and Lushan Ms7.0 earthquakes and found that the HVSRs move toward the low frequency when suffered nonlinearity. The velocity profiles are inverted using the HVSRs of weak S-wave motions, the theoretical transfer function and observed HVSRs are compared. The results show that the HVSR resembles the transfer function even in the circumstance of strong nonlinearity. **Fig 8** presents the theoretical spectral ratios (defined as TFs in [23], evaluated by the ratios between the horizontal motion at the surface and the horizontal motion of incidence waves at the basement) from 1-D simulation for the inverted soil models of 51SFB and 51WCW, which are located in Western China. The Figure shows the observed HVSRs, theoretical spectral ratios and theoretical borehole responses. Under the conditions of either weak (PGA about 10cm/s^2^) or strong motions (with PGA 560 cm/s^2^ for 51SFB and 960 cm/s^2^ for 51WCW), the HVSRs agree quite well with the theoretical spectral ratios, which illustrates that the HVSRs and the theoretical spectral ratios can still reveal the amplification of the site consistently even when velocity profiles change during severe earthquakes. Under the strong motion excitation, the nonlinear deformation process of soil will manifest itself in two ways in seismological observation: wave velocity decreases and resonance frequency will be therefore shifted downward. Simultaneously, increased dissipation will reduce soil amplification in the strong motion compared to the weak motion as shown in **Fig 8**. The theoretical borehole responses are linear results which indicate the site response. In the relatively low frequency range within *f*_0_ (about 7Hz for 51SFB and 10Hz for 51WCW), the theoretical borehole response can be ignored, the HVSR and transfer function agree quite well. In the relatively higher frequency range (>10Hz) where the theoretical borehole response can’t be ignored, the discrepancy between the HVSR and the transfer function is obvious.

## Conclusions

The observations of ground motion in two vertical arrays presented and analyzed here have provided fundamental data for the comparison of HVSR and TF. The discrepancy between HVSRs and TFs has been explored and interpreted by comparing vertical site response to theoretical and synthetic borehole or outcrop response. In addition, the applicability and conditions of using HVSR as TF have been studied. From what we have discussed above, we can come to the conclusion that the HVSRs from observed earthquakes resemble the TFs for horizontal components, the HVSR method can be used to determine predominant frequency. But discrepancy still exists in absolute amplitudes of HVSRs and TFs. The consistency of them depends on the vertical seismic response of the site, which is also frequency-dependent. The vertical site response can be explained by the P-wave amplification.Commonly, in the low-frequency range within *f*_0,_ the level of the vertical P-wave amplification is slight, while in high-frequency range near 2 *f*_0_ or larger, the P-wave amplification is obvious. Otherwise, the HVSRs and TFs are compared considering the site nonlinearity under severe earthquakes, the results show that in the frequency range in which the P-wave amplification can be ignored, the HVSRs and TFs are in good agreement in amplitude and spectrum.
